# Impact of HCV Infection on Diabetes Patients for the Risk of End-Stage Renal Failure

**DOI:** 10.1097/MD.0000000000002431

**Published:** 2016-01-22

**Authors:** Jyh-Chang Hwang, Ming-Yan Jiang, Yi-Hua Lu, Shih-Feng Weng

**Affiliations:** From the Division of Nephrology, Chi Mei Medical Center, Tainan, Taiwan (JCH, MYJ, YHL); Department of Hospital and Health Care Administration, Chia Nan University of Pharmacy and Science, Tainan, Taiwan (JCH, SFW); and Division of Medical Research, Chi Mei Medical Center, Tainan, Taiwan (SFW).

## Abstract

Both diabetes mellitus (DM) and hepatitis C virus infection (HCVI) are associated with chronic kidney disease (CKD). The aim of this study was to evaluate whether HCVI increases the risk of end-stage renal disease (ESRD) in patients with DM.

The National Health Insurance Research database of Taiwan was used to conduct this study. After excluding patients with a prior history of CKD, all patients with a first diagnosis of DM from January 1, 2000 to December 31, 2002 were enrolled. The patients who also had HCVI were defined as index cases (HCV group, n = 9787). A comparison cohort at a 1:1 ratio of random incident patients with DM without HCVI matched by age, sex, age at the diagnosis of DM, duration between the diagnosis of DM and the index date, and various comorbidities through propensity score matching were recruited (non-HCV group, n = 9787). The patients were followed until December 31, 2011.

The cumulative incidence rate of developing ESRD was significantly higher in the HCV(+) group than in the non-HCV group (*P* = 0.008). The incidence rate ratio (IRR) for the risk of ESRD was also significantly higher in the HCV(+) group (IRR: 1.44; 95% CI: 1.09–1.89) than in the non-HCV group, especially for those with a younger age (<50 years; IRR: 2.05; 95% CI: 1.22–3.45) and HCVI within 4 years after the diagnosis of DM (IRR: 1.85; 95% CI: 1.16–2.97). After adjusting for comorbidities in multivariate Cox proportional hazard regression analysis, HCVI (HR: 1.47; 95% CI: 1.11–1.93) was an independent factor for developing ESRD in the patients with DM. After starting dialysis for ESRD, the HCV(+) patients had a similar mortality rate to those without HCVI (*P* = 0.84).

HCVI increases the risk of developing ESRD in patients with DM, especially in younger patients and in those who develop HCVI sooner after a diagnosis of DM.

## INTRODUCTION

Diabetes mellitus (DM) is one of the most significant public health problems worldwide. Globally, 12% of health expenditure or USD 1330 per person were spent on DM in 2010.^1^ In Taiwan, the direct costs of healthcare for diabetic patients, including diabetic medications, clinic visits, and hospital admissions, account for 11.5% of the total national healthcare costs, and this is 4.3 times higher than the average cost of care for individuals without diabetes.^2^ Moreover, DM is the leading cause of end-stage renal disease (ESRD) in the United States, accounting for 36% of incident ESRD patients in 1990 and 44% in 2006.^3^ In Taiwan, DM-related ESRD also accounts for around 44% of all known cases of ESRD.^4^

Hepatitis C virus (HCV) infection not only causes chronic liver disease but can also lead to extrahepatic manifestations such as mixed cryoglobulinemia, lymphoproliferative disorders, and chronic kidney disease (CKD).^5^ Taiwan, with a 4.4% prevalence rate, is an endemic area for HCV infection.^6^ HCV infection is even more strongly associated with an increased risk of CKD compared to the traditional risk factors, and in a population-based retrospective cohort study, HCV-seropositive patients were more likely to develop ESRD than those who were HCV seronegative.^7^

Patients with DM superimposed with HCV infection represent a more complex situation, with both conditions potentially leading to renal failure via different pathways. However, whether or not HCV infection further increases the risk of uremia in DM patients is unknown. To date, very little research,^8^ especially by using large-scale analyses, has evaluated the impact of HCV infection on patients with DM with regards to developing ESRD. In this population-based study, we compared the incidence rate of ESRD in patients with DM with and without HCV infection in Taiwan from 2000 to 2002. The long-term survival of the patients with kidney failure who started maintenance dialysis in both groups was also compared. We further examined the additive effect of HCV infection on the final outcomes of the diabetic ESRD patients, since HCV infection is associated with higher morbidity and mortality rates and also has a negative effect on life expectancy.^9^

## MATERIALS AND METHODS

This study was conducted using data from the Longitudinal Cohort of Diabetes Patients (LHDB) in the National Health Insurance (NHI) program of Taiwan, which contains randomized selected data from patients (120,000 patients/per year) with newly diagnosed DM. The definition of DM in the LHDB is based on either of the 2 following conditions: *Inpatients*: at least 1 diagnosis of DM or a prescription for antidiabetes medication; *Ambulatory care*: at least 2 diagnoses of DM or at least 1 diagnosis of DM with a prescription for antidiabetes medication. The inclusion criteria of the patients in this study were those with a first diagnosis of DM according to the International Classification of Disease, Revision 9, Clinical Modification (ICD-9-CM) code 250 from January 1, 2000 to December 31, 2002. This study was conducted using a database without human participants. All of the data were analyzed anonymously, and the Research Ethics Committee of the Chi Mei Medical Center approved this study.

### Study Population

In addition to a new diagnosis of DM from January 1, 2000 to December 31, 2002, the inclusion criteria included a diagnosis of HCV infection (ICD-9-CM codes 07041, 07042, 07043, 07044, 07051, 07052, 07053, 07054, and V0262) according to a positive antibody test (HCV+). The patients with a history of CKD (ICD-9-CM codes 582, 583, 585, 586, and 588) before the index date were excluded. Those with human immunodeficiency virus infection (ICD-9-CM codes 042-044) and intravenous drug abusers (ICD-9-CM codes 305.90) were also excluded. A total of 9787 patients were enrolled as index cases and were followed up to December 31, 2011.

The date of a diagnosis of ESRD was defined as when the patients first started dialysis after suffering from HCV infection. All of these patients were maintained by regular dialysis for more than 90 days, and further received a catastrophic illness certificate with a code number of 585.

### Control Group

A control cohort was recruited at a 1:1 ratio of random incident DM patients without HCV infection (the non-HCV group) after excluding the patients with a previous history of CKD, with each control being matched by age, sex, age at the diagnosis of DM, duration between the diagnosis of DM to the index date, comorbidities including hypertension, coronary artery disease (CAD, ICD-9-CM codes 410-414), hyperlipidemia, gout, hepatitis B virus (HBV) infection, and liver cirrhosis using propensity score matching (the non-HCV control group, n = 9787). The index date of the HCV group was defined as the first date of a diagnosis of HCV infection, while the index date of the control group was defined as the year of the case's index date. The definitions of various comorbidities and the date of death were described in our previous study.^10^

### Statistical Analyses

The Student *t* test and χ^2^ test were used for comparisons of baseline categorical and continuous variables, respectively, between the patients with DM with and without HCV infection (Table 1). By estimating the incidence rate ratio (IRR) with Poisson regression,^10^ the risk of developing ESRD between the patients with DM with and without HCV infection was evaluated (Table 2). Cox proportional hazard analysis was further performed to analyze the risk factors for developing ESRD during the follow-up period (Table 3). The cumulative incidence rates for developing ESRD and the actuarial survival rates of the 2 groups were determined by the Kaplan–Meier method. A log rank test was applied to compare the difference between 2 survival curves after the development of ESRD for the patients on maintenance dialysis. A *P*-value of less than 0.05 was considered to be statistically significant. All of the analyses were conducted using SAS statistical software (version 9.3.1, SAS Institute, Cary, NC).

**TABLE 1:**
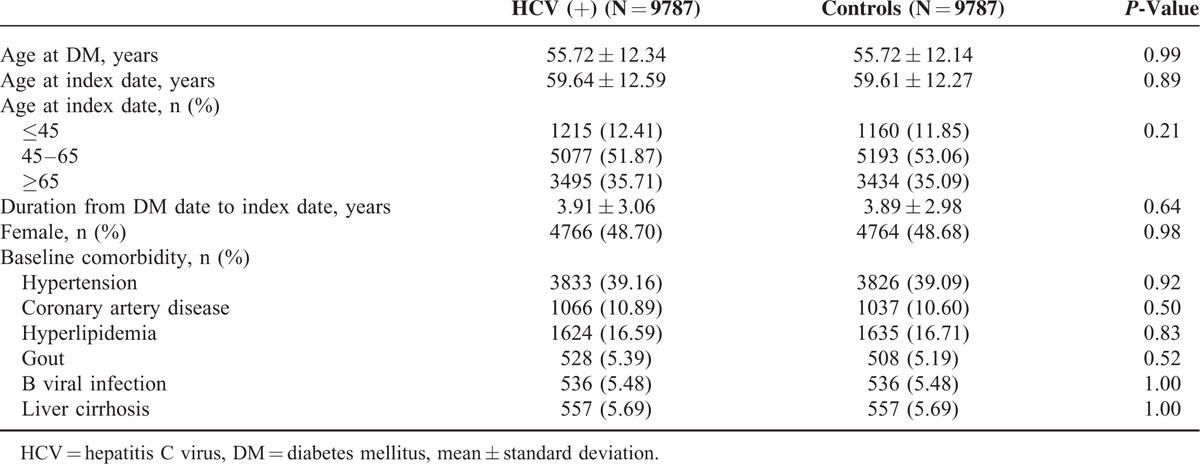
Differences in Baseline Demographic Data Between the Patients With DM With and Without HCV Infection

**TABLE 2:**
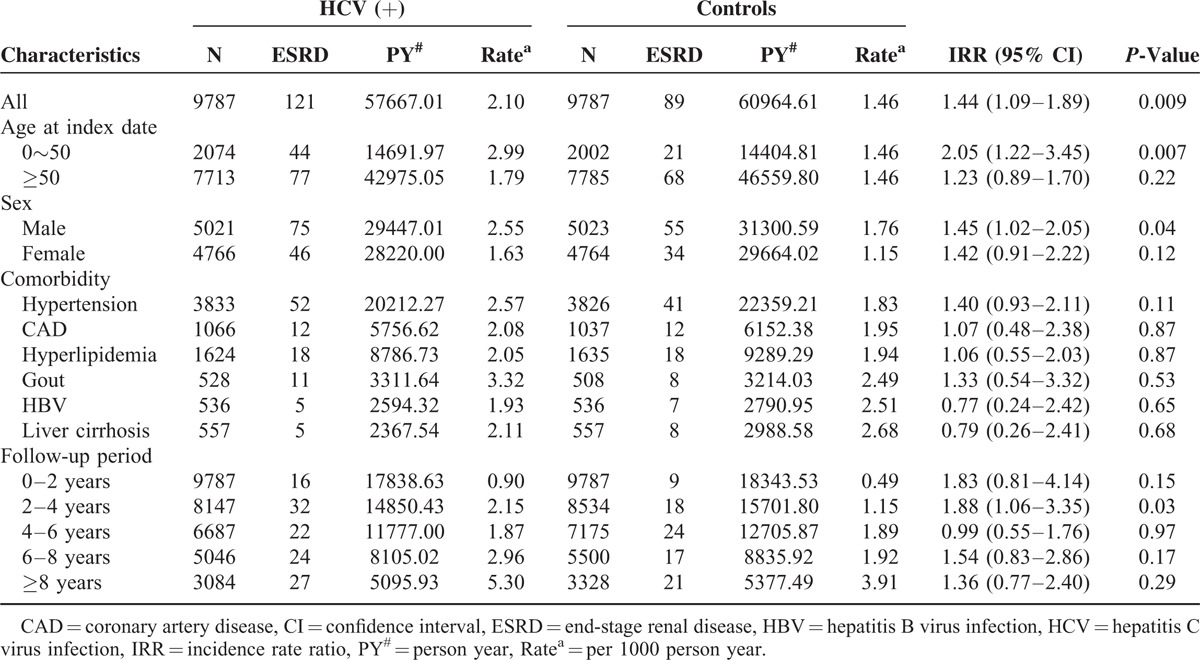
Subgroup Analysis of the Risk of ESRD by Age, Sex, Comorbidity and Follow-Up Duration

**TABLE 3:**
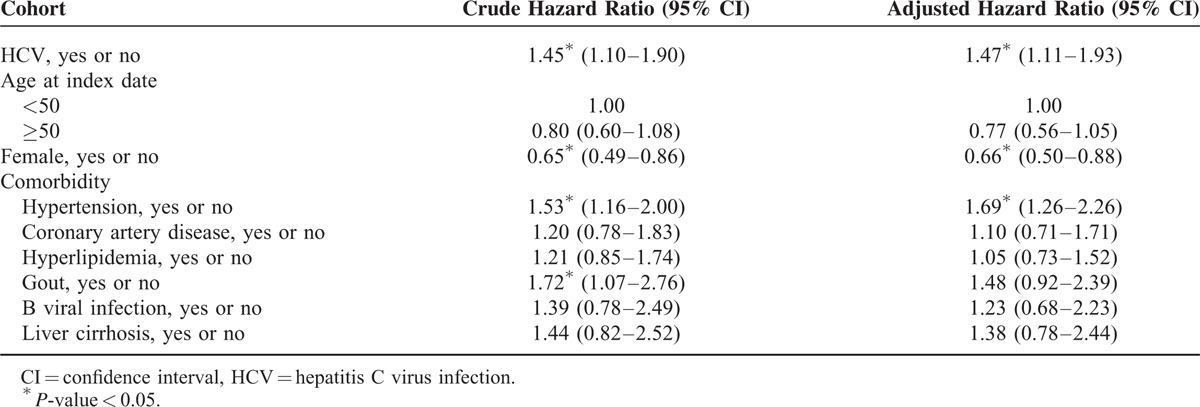
Cox Proportional Hazard Regression Analysis for the Effect of HCV Infection on the Risk of ESRD

## RESULTS

### Differences in Baseline Demographic Data Between the Patients With DM With and Without HCV Infection

The patients with HCV infection had similar percentages of hypertension, CAD, hyperlipidemia, HBV infection, liver cirrhosis, and gout to those without HCV infection (Table 1). In addition, both groups also had similar index dates and ages at the index date.

### Subgroup Analysis of the Risk of ESRD by Age, Sex, Comorbidity, and Follow-Up Duration

Compared to the control group, the HCV(+) patients tended to have a higher risk of ESRD (IRR: 1.44; 95% CI: 1.09–1.89), especially in those under the age of 50 years (IRR: 2.05; 95% CI: 1.22–3.45). After stratifying by sex, males (IRR: 1.45; 95% CI: 1.02–2.05) with HCV infection were at a higher risk of developing ESRD than the male controls (Table 2). When stratified by comorbidities, the HCV(+) patients with hypertension, CAD, hyperlipidemia, and gout had similar risks of developing ESRD as the controls (Table 2). In addition, the earlier the HCV infection after the diagnosis of DM, and especially from 2 to 4 years (IRR: 1.15, 95% CI: 1.06–3.35), the higher the risk of ESRD.

### The Incidence Rate of ESRD and Survival Analysis After Dialysis

**FIGURE 1:**
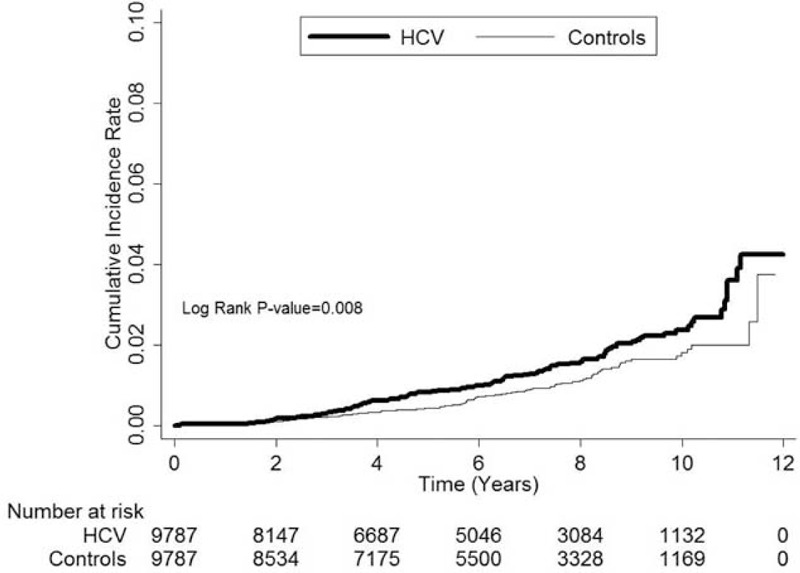
The cumulative incidence rate for developing end-stage renal disease was significantly higher in the patients with hepatitis C virus (HCV) infection compared to the control group (*P* = 0.008).

**FIGURE 2:**
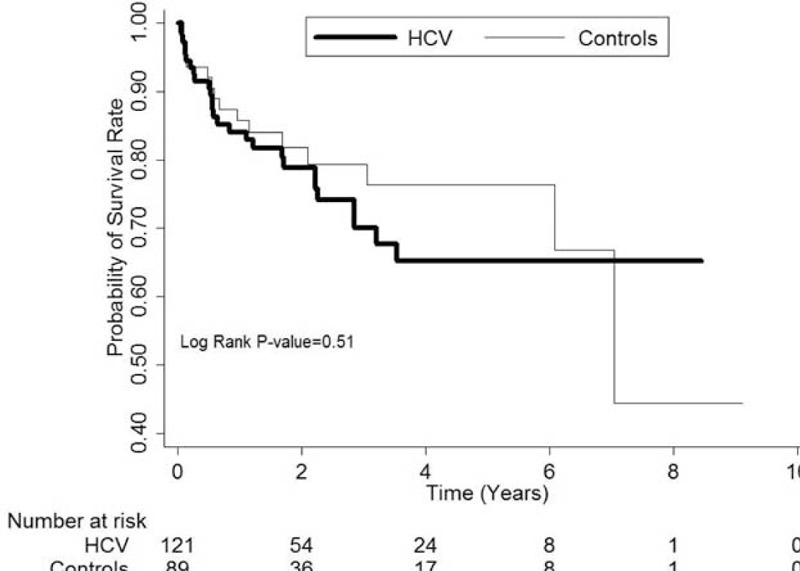
After developing terminal uremia, the patients with hepatitis C virus (HCV) infection had a similar cumulative survival rate to those without HCV infection (Controls, *P* = 0.51).

The cumulative incidence rate for developing ESRD was significantly higher in the HCV(+) group compared to the control group (*P* = 0.008, Figure 1), especially 2 to 4 years after the diagnosis of DM. After developing ESRD, the HCV(+) patients had a similar cumulative survival rate to the DM patients without HCV infection (*P* = 0.51, Figure 2).

### Cox Proportional Hazard Regression Analysis for the Effect of HCV Infection on the Risk of ESRD

After adjusting for sex, age at index date and various comorbidities including hypertension, CAD, hyperlipidemia, HBV infection, gout and liver cirrhosis, multivariate Cox proportional hazard regression analysis showed that HCV(+) (HR: 1.47; 95% CI: 1.11–1.93) was a significantly risk factor for the development of ESRD (Table 3).

## DISCUSSION

The main finding of this study is a higher incidence rate of ESRD in patients with DM superimposed with HCV infection. In other words, HCV infection appears to increase the risk of developing ESRD in patients with DM. In addition, the earlier the HCV infection, especially between 2 and 4 years after the diagnosis of DM, the higher the risk of developing ESRD. Furthermore, compared to the female patients, the male patients had a higher risk of developing ESRD after HCV infection, and the patients with more comorbidities including hypertension, CAD, hyperlipidemia, and gout also had a higher risk of developing ESRD. After adjusting for these comorbidities, HCV infection was still a significant factor leading to uremia in the patients with DM. However, after starting maintenance dialysis for ESRD, the patients with DM and HCV infection did not have a higher long-term mortality rate compared to their HCV(−) counterparts.

Diabetes is the most important risk factor for CKD and is also the leading cause of ESRD in the United States, accounting for 44% of incident cases of ESRD in 2010.^11^ The prevalence of any stage of CKD in patients with type 2 DM (T2DM) was reported to be 27.9% in a national cross-sectional study in Spain.^12^ In Taiwan, DM also accounts for 25% to 33% of chronic dialysis patients.^4^

Chronic HCV infection has also been associated with significantly higher incidence and prevalence rates of CKD.^13,14^ The prevalence of HCV seropositivity increases with advanced CKD stage.^15^ In addition, infection with HCV has also been reported to be significantly positively associated with proteinuria.^16^ In a retrospective cohort study involving over 470,000 adult veterans, patients with HCV infection were more likely to develop ESRD than HCV-seronegative patients.^17^ Moreover, in patients with any stage of CKD, the presence of HCV infection has been reported to be associated with a 30% higher risk of ESRD.^18^

Patients with chronic HCV infection have been reported to have insulin resistance and a higher prevalence of diabetes.^19^ HCV infection activates the mTOR/S6K1 pathway which inhibits the function of insulin receptor substrate 1, downregulates the expression of glucose transporter, and upregulates the expression of the gluconeogenic enzyme PCK2, both of which are known to induce insulin resistance and increase blood glucose levels.^20^ Insulin resistance and hyperinsulinemia increase the intrarenal production of insulin-like growth factor-1 (IGF-1) and transforming growth factor beta (TGF-β), both of which promote renal cell proliferation. Insulin also upregulates angiotensin II type 1 receptors in mesangial cells, thereby intensifying the adverse effects of angiotensin II on the kidneys.^21^ Moreover, insulin resistance and hyperinsulinemia are associated with excessive local production of endothelin-1, decreased endothelial production of nitric oxide and increased oxidative stress.^22^

Insulin treatment has recently been shown to decrease the response to cardiac resynchronization therapy,^23^ which may be attributed to an alteration in the relationship between mitogenic and metabolic pathways. It remains to be elucidated whether insulin aggravates the impact of HCV infection on patients with DM with regards to the progression of renal failure.

In contrast, a meta-analysis showed a higher risk of acquiring HCV infection in patients with T2DM than in those without DM.^24^ The risk of a nosocomial HCV infection in hospitalized diabetic patients has been reported to be associated with frequent transfusions, blood sampling, intravenous drug use, being treated in a hepatogastroenterology center and a high number of previous admissions after the onset of diabetes.^25^ Disturbances in humoral and cellular immunity in patients with DM may contribute to the high risk of HCV infection. Owing to an immunosuppressed status, T2DM may be involved in impaired HCV clearance. In addition, HCV replication may be enhanced by hyperinsulinemia in patients with insulin resistance and diabetes.

Complications related to macro- and microangiopathies in patients with DM and hypertension can cause progressive background nephrosclerosis. Immunologically, HCV infection is associated with a variety of chronic glomerulonephritis, and to some degree leads to a negative impact on the severity of diabetes-related nephropathy. Moreover, adverse effects associated with the treatment regiment of HCV infection, including herbal drugs, nephrotoxicity caused by diagnostic procedures for chronic C hepatitis and sequelae of HCV-related liver cirrhosis may also worsen the deterioration of renal function. All of these factors can cause multiple episodes of kidney injury which aggravate background chronic fibrosis. Ultimately, HCV infection accelerates the deterioration of renal function and causes ESRD.

A younger age and earlier HCV infection after the diagnosis of DM were 2 important risk factors associated with the development of ESRD in this study. Cardiovascular death is traditionally the predominant cause of mortality in older patients; however, a longer life span means that younger patients with DM have a longer time period to develop renal injuries and terminal uremia. Furthermore, the earlier the HCV infection, the longer the period of additional interaction between the 2 disease entities, which then eventually may cause more severe renal injuries and ESRD.

After renal failure, the HCV(+) patients on dialysis had a similar cumulative survival rate to those without HCV infection, even though HCV infection has been reported to lead to a poorer survival compared to those without HCV infection. This may be due to an earlier diagnosis of HCV infection as well as a more adverse impact of serious uremic complications during the follow-up period.

In addition to the risk of renal failure, DM is also associated with a higher incidence of hepatocellular carcinoma (HCC) in patients with chronic liver disease,^26^ especially in those with a younger age and in those who smoke and use insulin.^27^ The presence of DM has also been reported to increase the risk of mortality due to HCC.^28^ However, increased coffee consumption seems to reduce the risk of HCC and chronic liver disease.^29^

There are 3 main strengths to this study. First, it was a large-scale study including 9787 patients with DM and HCV infection with 12 years of follow-up. Second, differences in cumulative incidence rates were examined yearly to observe the timing of the differences between the HCV(+) and the control groups. Third, the risk of ESRD was also analyzed after stratification by sex, age, timing of HCV infection and comorbidities.

There are also several limitations to this study. First, the stage of HCV infection at diagnosis and the virologic status of HCV infection were not recorded, and the renal etiology of the HCV infection could not be clarified. Second, baseline residual renal function and the state of proteinuria were not evaluated. We assumed that the renal function of both groups was equal, as all of the patients with a history of CKD were excluded before enrollment. The similarity of the incidence risk for ESRD in the first 2 years between the groups may also suggest a similar baseline renal function status. Third, we were unable to trace the details of blood glucose and pressure control, both of which are important given that the severity of hyperglycemia and hypertension are related to an accelerated deterioration of renal function. We assumed relatively similar blood glucose and pressure control between the 2 groups. Finally, we did not record the cause of death. However, these omissions are not expected to have affected our results of the estimate of the difference in the incidence of ESRD in patients with DM and with and without HCV infection in Taiwan.

In conclusion, patients with DM and HCV infection tended to have a significantly higher incidence rate of ESRD, particularly in those who were infected by HCV at a younger age and within 4 years after the diagnosis of DM. HCV infection increases the risk of DM-related nephrosclerosis, and a longer period of interaction between the 2 disease entities significantly worsened the decline in renal function. In addition to tight control over blood glucose and pressure to slow the progression of diabetic nephrosclerosis, it is important to prevent HCV infection in patients with DM, and to initiate early effective treatment for HCV if infected. However, further studies are needed to confirm the mechanism by which HCV infection accelerates renal failure in patients with DM.
